# The National Dementia Care Collaborative: Outcomes From Year 1

**DOI:** 10.1093/geroni/igaf122.828

**Published:** 2025-12-31

**Authors:** Gary Epstein-Lubow, Kristin Haggerty, Maria Avalos, Madelyn Johnson, Rebecca Stoeckle

**Affiliations:** Education Development Center, Waltham, Massachusetts, United States; Education Development Center, Waltham, Massachusetts, United States; Education Development Center, Waltham, Massachusetts, United States; Education Development Center, Waltham, Massachusetts, United States; Education Development Center, Waltham, Massachusetts, United States

## Abstract

The National Dementia Care Collaborative (NDCC) aims to improve access to and successful implementation of evidence-based comprehensive dementia care. NDCC provides a common learning platform for health systems and other provider organizations implementing or interested in implementing a proven model of comprehensive dementia care. Evidence-based model teams participating with NDCC include the Aging Brain Care (ABC) Program from Indiana University, the Alzheimer’s and Dementia Care (ADC) Program from UCLA, the Benjamin Rose Institute’s Care Consultation (BRI-CC), the Care Ecosystem from UCSF, Maximizing Independence (MIND) at Home from Johns Hopkins University, and the Integrated Memory Care (IMC) program from Emory University, This session will provide descriptions of implementation strategies that are common across these comprehensive dementia care models as well as examples of specific implementation strategies that are unique to one or more model(s). Outcomes will be described regarding the number of new implementations of an evidence-based comprehensive dementia care program in health systems and community-based organizations participating in the CMS GUIDE Model and external to it; this included 51 total new programs in 2024. New emphasis on growing the interest in dementia care models in value-based care, Medicare Advantage, and commercial health plans will also be described.

